# Supplementary material for: The impact of mass drug administration expansion to low onchocerciasis prevalence settings in case of connected villages
Source: PLoS Negl Trop Dis. 2021 May 12;15(5):e0009011. doi: 10.1371/journal.pntd.0009011 (PMC8143415; doi:10.1371/journal.pntd.0009011)
Supplement: S1 Text — Table A in S1 Text. PRIME-NTD checklist guiding principles for policy relevant modelling. Fig A in S1 Text. Spill-over equilibrium prevalence as influenced by the type of connection between two villages. Fig B in S1 Text. Spill-over equilibrium prevalence as influenced by the local ABRs of two connected villages. Fig C in S1 Text. Spill-over prevalence as influenced by the relative size of the two villages. Fig D in S1 Text. Spill-over prevalence in case of three villages. Fig E in S1 Text. The individual variation in time spent outside the home village (as controlled by the model input parameter M, see the Methods section) and the resulting spill-over of prevalence. Fig F in S1 Text. Impact of MDA strategy on the probability of onchocerciasis elimination for different geographical settings. Fig G in S1 Text. Impact of MDA strategy in scenarios with three villages. Fig H in S1 Text. Impact of MDA strategy in scenarios with different MDA coverages. Fig I in S1 Text. Impact of MDA strategy in scenarios with different individual variation in the time spent outside the home village. Fig J in S1 Text. Decrease in the number of years of MDA required for 90% onchocerciasis elimination probability, by treatment implemented throughout also in the spill-over prevalence village(s) rather than just in the higher prevalence village (parameter sensitivity scenarios). Fig K in S1 Text. Decrease in the number of years of MDA required for 80% or 99% onchocerciasis elimination probability, by treatment implemented throughout also in the spill-over prevalence village(s) rather than just in the higher prevalence village. Fig L in S1 Text. Decrease in the number of years of MDA required for 90% onchocerciasis elimination probability, by treatment implemented throughout also in the spill-over prevalence village(s) rather than just in the higher prevalence village (with three village scenarios per village comparisons). Fig M in S1 Text. Impact of MDA duration on onchocerciasis elimination [file pntd.0009011.s001.pdf]

S1 Text

***Supplement to:* The impact of mass drug administration expansion to low onchocerciasis prevalence settings in case of connected villages**

Anneke S. de Vos[1,]*, Wilma A. Stolk[1], Luc E. Coffeng[1,#], Sake J. de Vlas[1,#]

1. Department of Public Health, Erasmus MC, University Medical Center Rotterdam, Rotterdam, The Netherlands

#. These authors contributed equally to the work

* a.s.devos@erasmusmc.nl

Detailed methods with mathematical formulae

Model extension. For the current analysis, we have adapted the ONCHOSIM version that is described in our paper on the influence of assortative mixing [9], which is implemented in the program *R* [16]. As stated in our main text, we have adapted the model to now include multiple villages, that can be targeted separately with MDA. Each village has their own fly population ('cloud'). We allow for different levels of contact between the villages, either by regular human or by fly movement (see Main text Fig 1 for a visual description of the model). Here we explain in full detail how we have achieved this model adaptation.

Let *j* be the index for a human subpopulation and its associated vector cloud. Ex_{ij} represents the annual exposure of individual i to fly-bites from cloud j and is given by: $Ex_{ij} = ABR_j \cdot Exi_i \cdot Exa(a_i) \cdot Ext_{ij}$. Here, ABR_j represent the yearly number of fly bites that an average adult experiences if spending all time in village *j*. Exi_i represents an individual scaling factor (i.e. impact of behaviour, occupation, attractiveness to blackflies), this factor is drawn from a gamma distribution with both shape and rate k, and thus mean equal to 1. Individual exposure is further determined by the age a_i of the individual, through age-specific relative exposure $Exa(a_i)$ (Exa increases linearly until age 20, from 0 to 1). Finally – new in this model version – the term Ext_{ij} is the fraction of time an individual i spends in village *j*. The per individual definition of the ABR_j does imply that with more individuals, including visitors from another village, the total number of bites in an area will be larger. Since we presently allow only stable transit of humans or flies between villages (i.e. constant throughout a simulation), however, we implicitly also assume dynamically stable fly population sizes.

This model version works with monthly time-steps. For simplicity, seasonality in fly densities is here not included. Monthly mf uptake from the humans to the flies, $lu(t)_{up}$, is calculated per cloud, as $lu_j(t)_{up} = \sum_i u\big(sl_i(t)\big) \cdot Ex_{ij}/12$, where $u\big(sl_i(t)\big)$ is a density-dependent function of $sl_i(t)$, the larval density in the skin of host i. In a next step we allow for the direct movement of (infected) vectors. The L1 stage larval load per cloud is kept track of in the vector $\boldsymbol{lu(t)}$, calculated per time step as $\boldsymbol{lu}(t) = \boldsymbol{lu}(t)_{up} \cdot \boldsymbol{V}$, where $\boldsymbol{lu}(t)_{up}$ is the matrix of the $lu_j(t)_{up}$, and $\boldsymbol{V}$ is a matrix that defines the amount of movement from cloud to cloud (i.e. $V_{j_f j_t}$ is the fraction of flies moved from cloud j_f to cloud j_t at the time onchocerciasis larvae have reached their infectious stage).

The population level force of infection is calculated as $foi(t) = \sum_j(\, lu_j(t) \,) \cdot \zeta \cdot \nu$, where ν is the probability for larvae to develop to the infective L3 stage within the host fly, and ζ is the probability for an L3 larva injected into a new human host to successfully develop into an adult

worm. Note here the implicit assumption that movement between villages does not impact on the probability for infected flies to cause infection, i.e. their rate of biting humans stays constant if they move. As we assume independence of the per larva infection probabilities, a Poisson process governs the total resulting number of new infections lr_t in the population: $lr_t \sim \text{Pois}(\,foi(t)\,)$.

The fraction of all new infections generated by each cloud j is calculated as $lrf_j(t) = lu_j(t) / \sum_j(\, lu_j(t)\,)$. New infections are then distributed randomly over all individuals, with the per infection probability for a human to obtain an infection $p_i = \sum_j \;(\, lrf_j(t) \cdot Ex_{ij} \,/\, \sum_i Ex_{ij}\,)$. That is, an individual's probability to obtain a worm from a certain cloud is a linear function of the fraction of all bites from this cloud received by the individual i.

A human's home village V_i is assigned at birth, with probability p_j to be assigned village j ($\sum_j p_j = 1$) (so for example in case of two equal sized villages, $p_1 = p_2 = 0.5$). Subsequently an individual's time per village is drawn from the Dirichlet distribution (which is a multivariate generalization of the beta distribution): $Ext_{ij} \sim Dir(Ext_{V_i} / M)$, where Ext_{V_i} is a vector with the mean fraction of time that individual i living in village V_i spends in each village j in the area, and M is a scalar of the variance over this mean (larger M gives more variance). Note, in our analyses, the mean villager sojourn time is always chosen to be highest for the home village, although due to random variation (generated from the Dirichlet distribution), some individuals might still spend most of their time in a neighbouring village, for example where they work, rather than in their home village. Their infection status still counts for their home village prevalence level, and the MDA implementation status of their home village will determine whether or not they can receive drugs.

Model analyses. In this study we consider two or three villages, each consisting of around 400 individuals. We perform 100 runs per MDA setting, and the 100 initiating random seeds are kept constant for each setting, i.e. the starting conditions before MDA implementation are the same, which reduces the effect of randomised model events on the estimated impact of MDA implementation.

Inspection of runs showed that at 100 years post-MDA, onchocerciasis prevalence in the area has always either recovered to (near) pre-MDA levels, or it has become 0. We increased the duration of MDA by single years until reaching elimination of onchocerciasis at 100 years post-MDA in all 100 runs, and also lowered the duration until reaching 0% elimination. The number of years of MDA needed for 90% probability of elimination is then estimated by fitting a logistic regression model to the proportion of simulations that achieved elimination ($P_{elimination}$) for each MDA history, using splines. We define $log\left(\frac{P_{elimination}}{1-P_{elimination}}\right) = b_0 + \; b_1 \cdot M_1 + b_2 \cdot M_2 + b_3 \cdot M_3$, where M_v is the number of MDA rounds in village *v*, fitted in *R* by function glm(), setting family = binomial, and allowing splines with three knots for each predictor M (using the function "ns()" from the R package "splines"). In the two geographical settings where each combination $M_1 \leq \; M_2$ was considered, we also include the interaction of these MDA durations in our model fit; $log\left(\frac{P_{elimination}}{1-P_{elimination}}\right) = \; b_0 + b_1 \cdot M_1 + b_2 \cdot M_2 + b_4 \cdot M_1 \cdot M_2$, again including splines.

Policy relevant modelling. Table A provides a description of how we adhere to the five principles of the NTD Modelling Consortium.

Table A. PRIME-NTD checklist guiding principles for policy relevant modelling*

Principle	What has been done to satisfy the principle?	Where in the manuscript is this described?
1. Stakeholder engagement	For this more theoretical paper, stakeholders were not directly involved. However, previous discussions with policy makers, on the possibility of setting a prevalence threshold for implementation of MDA, did inspire and guide the model analyses.	In the Discussion section.
2. Complete model documentation	Described in detail in previous open access publications, with adaptation described in the current paper, and model source code included.	In the Methods section and S1 Text and S1 Model Code.
3. Complete description of data used	Parameters in the model rely on quantification from previous published studies.	Particular previous publications are referred to in the Methods section.
4. Communicating uncertainty	Limitations, specifically in the lack of knowledge on how stable low prevalence settings are actually sustained, are thoroughly discussed.<br><br>Stochastic uncertainty in simulated prevalence measurements is presented, and univariate sensitivity of model outcomes to several model parameters was performed.	Discussion section.<br><br>Results section and the Supporting information: uncertainty is included in main text Figs 2-4 and here in Figs A-E, sensitivity is explored in Figs E and H-K.
5. Testable model outcomes	Model outcomes include future mf trends, time to elimination, and the probability of elimination in a wide range of settings.	Results section.

* The Policy-Relevant Items for Reporting Models in Epidemiology of Neglected Tropical Diseases (PRIME-NTD) table, as described in: Behrend MR, Basáñez MG, Hamley JID, et al. Modelling for policy: The five principles of the neglected tropical diseases modelling consortium. *PLoS Negl Trop Dis*. 2020.

Further connectivity effects on prevalence

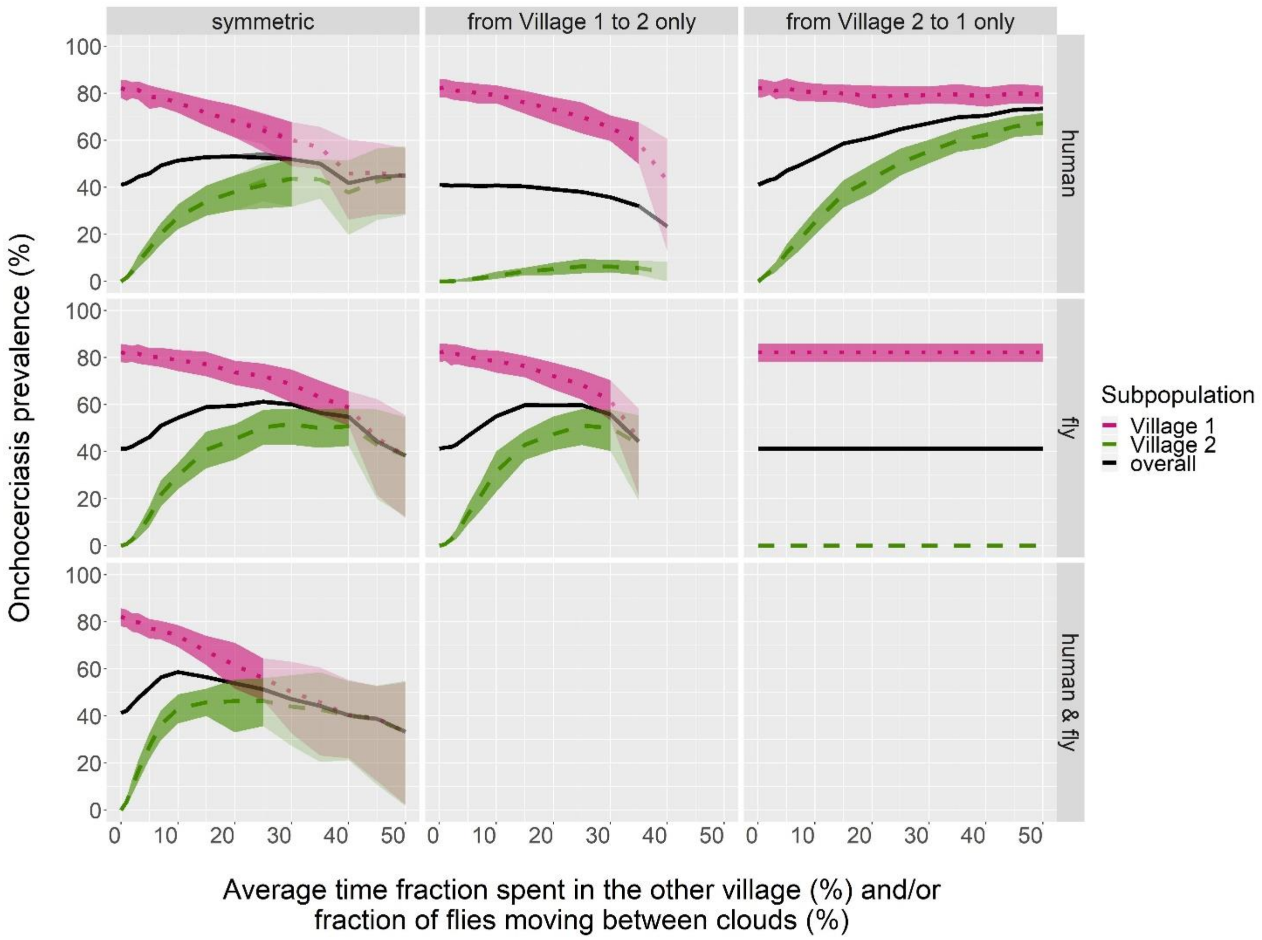

Fig A. Spill-over equilibrium prevalence as influenced by the type of connection between two villages. ABR Village 1 = 16,000; ABR Village 2 = 4,000. Movement is either symmetric, i.e. on average as many inhabitants or flies travel to Village 1 from Village 2 as vice versa, or travel occurs only by those from one of the two villages. Bands represent the 80% inter-decile range over the aggregate of monthly data-points for all runs. Transparency shows where 1-20 of the 40 runs ended with onchocerciasis eliminated; mean and variance were then calculated over remaining runs only, and lines are cut off where >20 runs had onchocerciasis disappear. Prevalence is among those aged 5+ years.

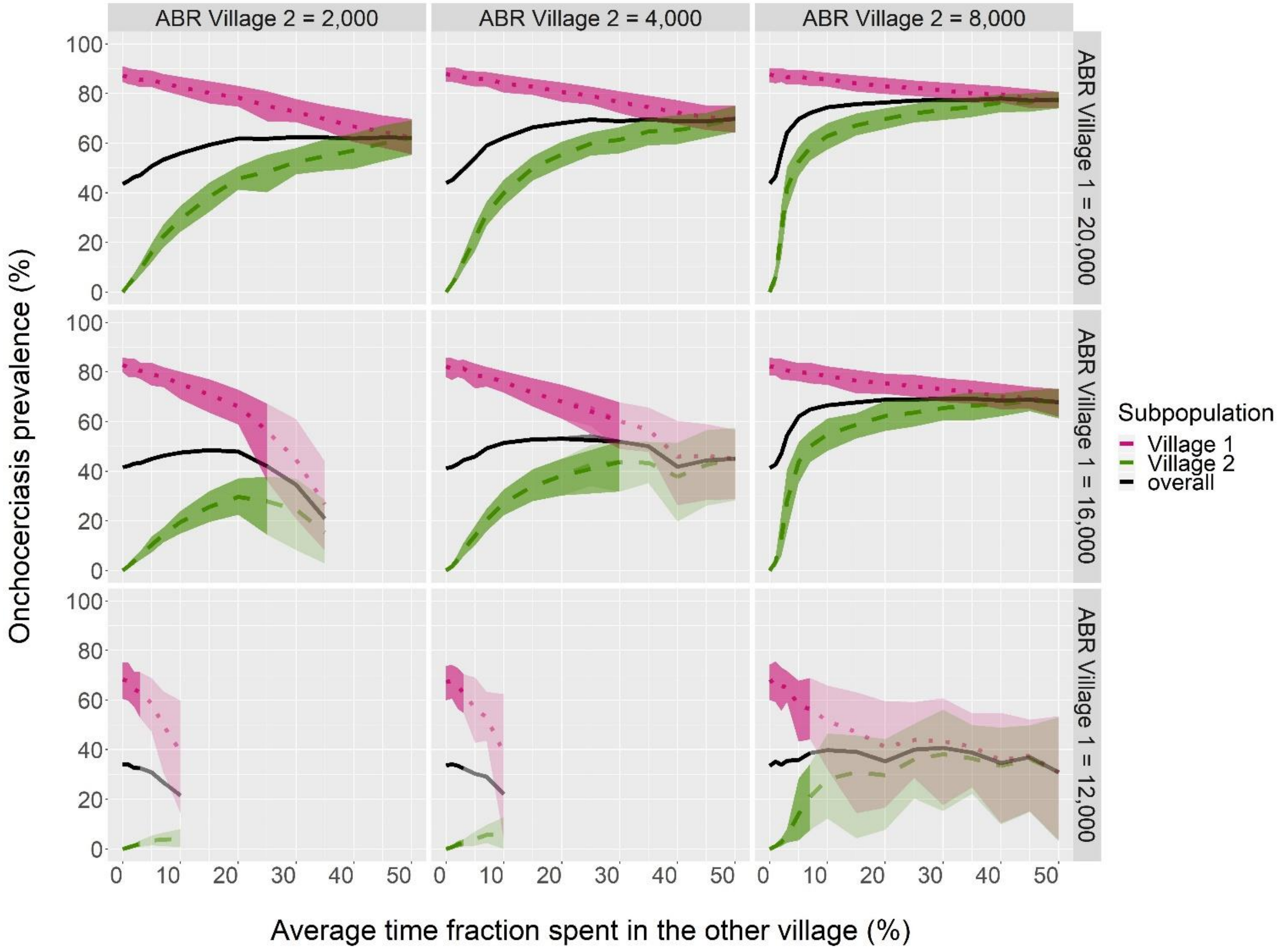

Fig B. Spill-over equilibrium prevalence as influenced by the local ABRs of two connected villages. Movement is human and symmetric between the villages. Bands represent the 80% inter-decile range over the aggregate of monthly data-points for all runs. Transparency shows where 1-20 of the 40 runs ended with onchocerciasis eliminated; mean and variance were then calculated over remaining runs only, and lines are cut off where >20 runs had onchocerciasis disappear. Prevalence is among those aged 5+ years.

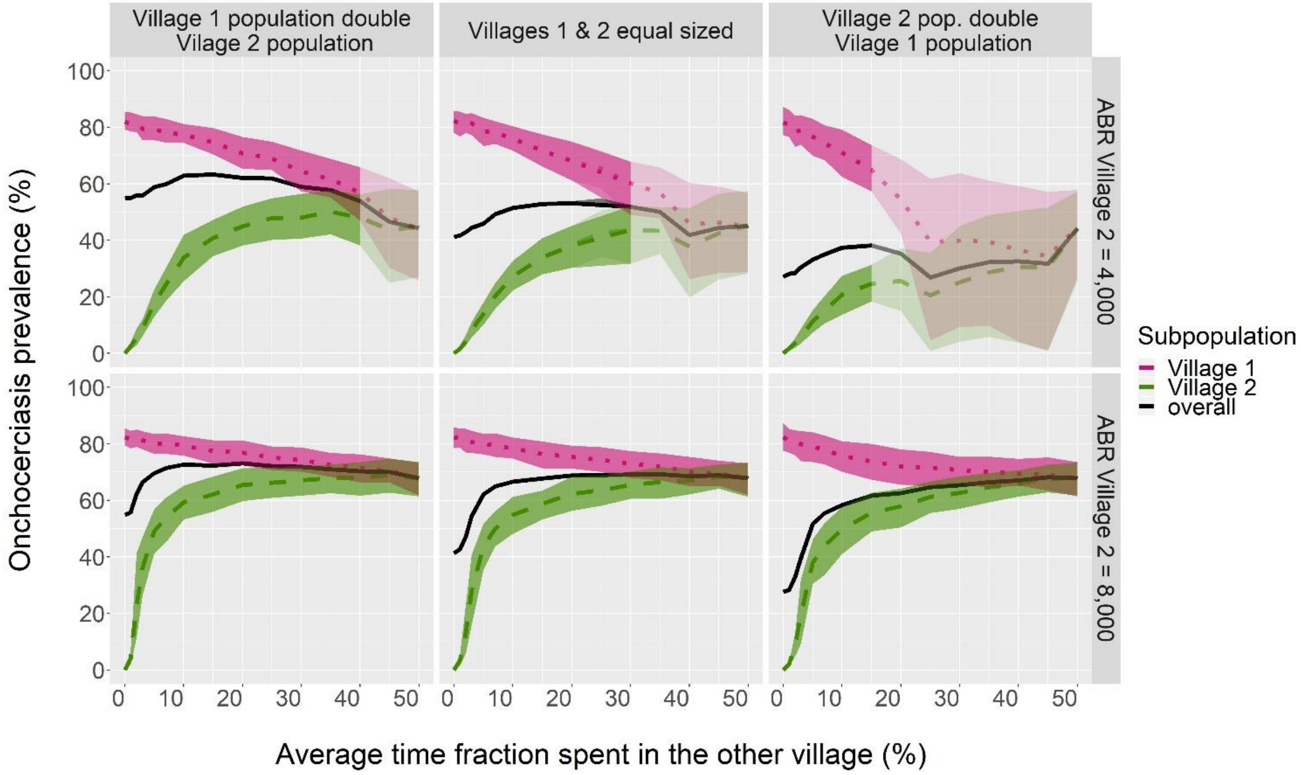

Fig C. Spill-over prevalence as influenced by the relative size of the two villages. Total population size is around 800, left column ⅔d, middle ½ and right column ⅓d of this population lives in Village 1. ABR Village 1 = 16,000. Movement is human and symmetric. Bands represent the 80% inter-decile range over the aggregate of monthly data-points for all runs. Transparency shows where 1-20 of the 40 runs ended with onchocerciasis eliminated; mean and variance were then calculated over remaining runs only, and lines are cut off where >20 runs had onchocerciasis disappear. Prevalence is among those aged 5+ years.

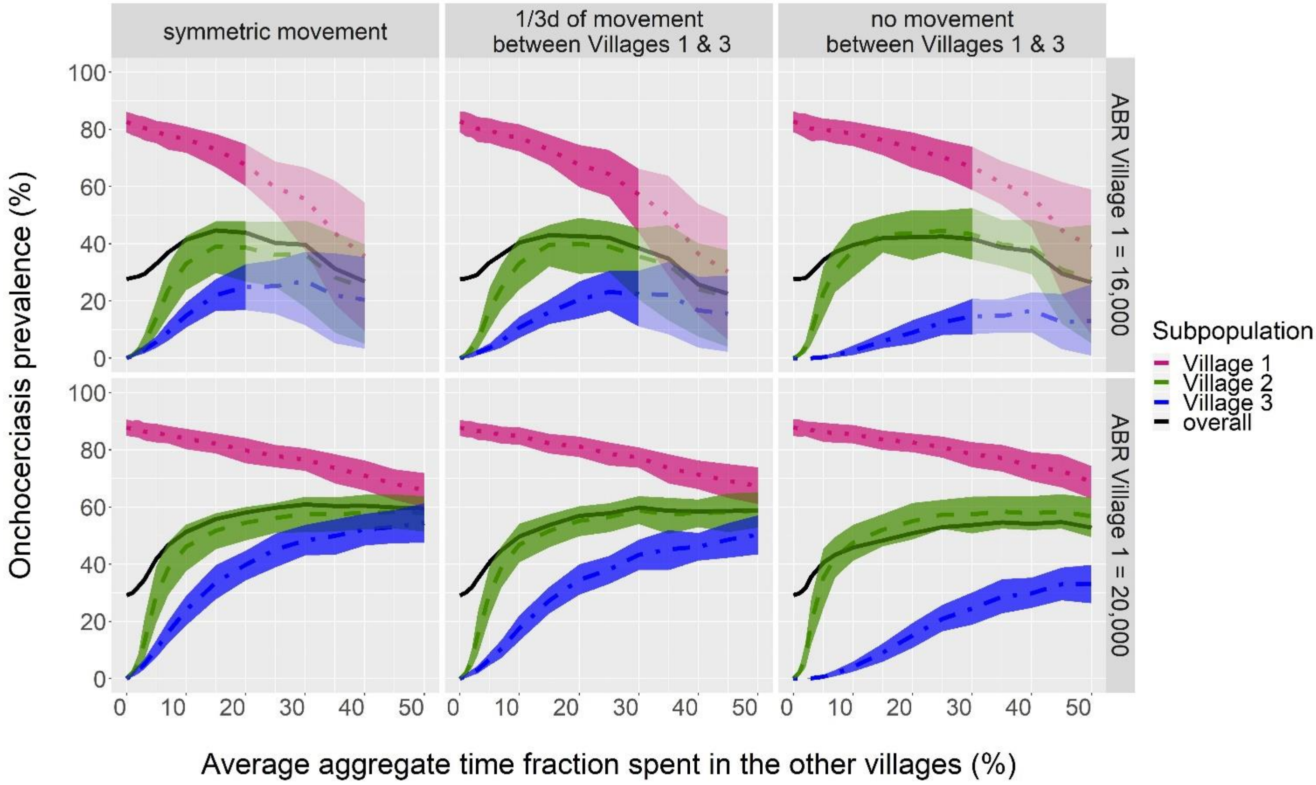

Fig D. Spill-over prevalence in case of three villages. ABR Village 2 = 8,000, ABR Village 3 = 4,000. Movement is human, and is either assumed to be symmetric between the three villages, with each non-home village visited equally on average, or Village 2 is assumed to be geographically in the middle of the two other villages. In the latter case either the villagers from Village 1 and 3 do visit each other's location but go to Village 2 twice as often as they visit the village further away (middle column), or the inhabitants from Village 1 and Village 3 do not travel to each other at all (right column). Bands represent the 80% inter-decile range over the aggregate of monthly data-points for all runs. Transparency shows where 1-20 of the 40 runs ended with onchocerciasis eliminated; mean and variance were then calculated over remaining runs only, and lines are cut off where >20 runs had onchocerciasis disappear. Prevalence is among those aged 5+ years.

In case all villages are equally connected to each other, the prevalence patterns over the rate of human exchange between the villages is fairly similar to the patterns shown before for the two village settings (see Figs A-C). If Village 3 is only indirectly connected to Village 1, however, through Village 2, then prevalence in Village 3 rises much more slowly with the human exchange rate between the villages.

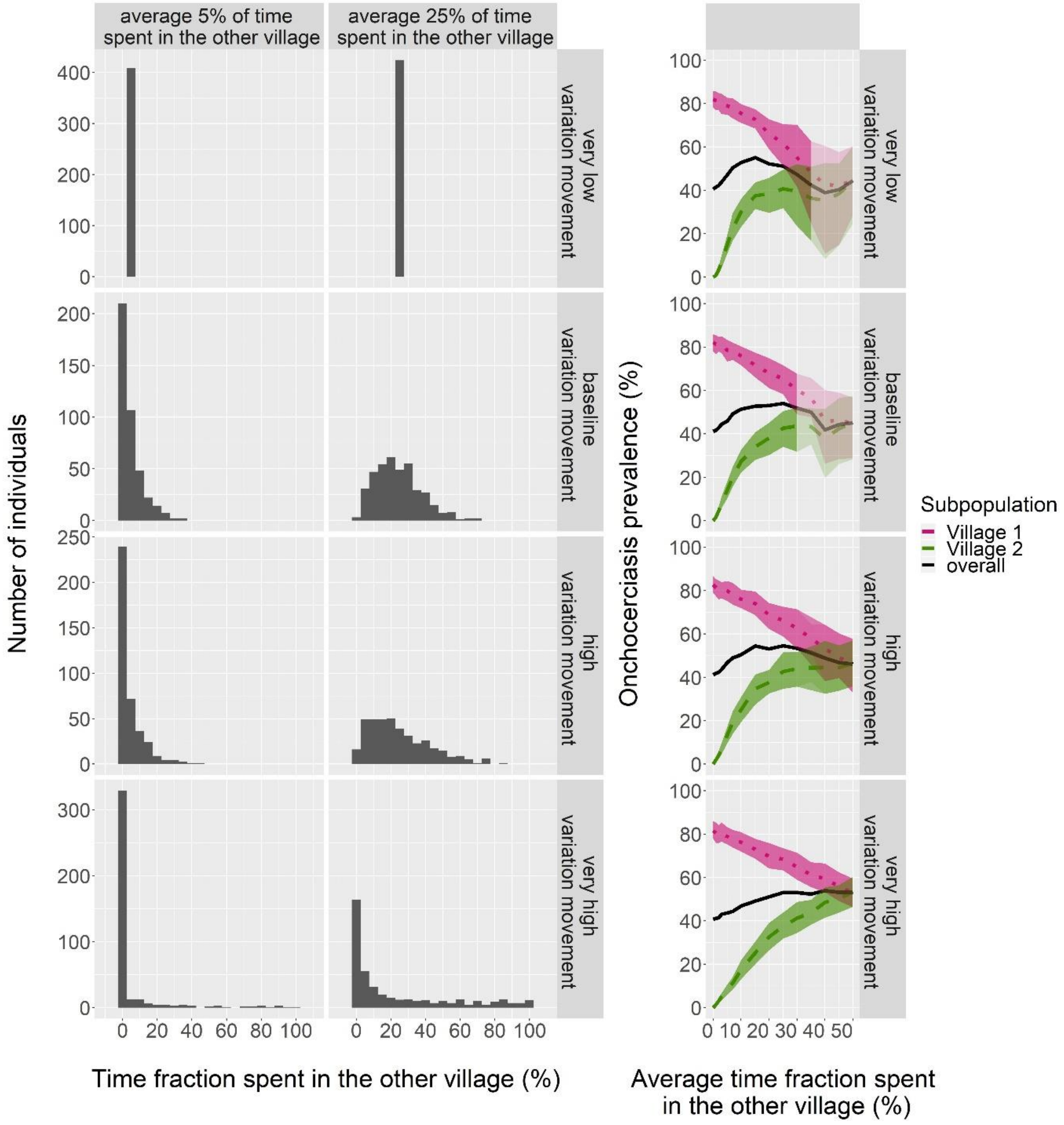

Fig E. The individual variation in time spent outside the home village (left columns, as controlled by the model input parameter M, see the Methods section) and the resulting spill-over of prevalence (right column). The histograms are based on 400 randomly drawn individuals, i.e. the approximate village size in most of our analyses. Variation is nearly absent (top, M = 0,0001), at base-line (second row, M = 0.1), high (third row, M = 0.2), or very high (bottom row, M = 1). ABR Village 1 = 16,000; ABR Village 2 = 4,000. Movement is human and symmetric. Bands represent the 80% inter-decile range over the aggregate of monthly data-points for all runs. Transparency shows where 1-20 of the 40 runs ended with onchocerciasis eliminated; mean and variance were then calculated over remaining runs only, and lines are cut off where >20 runs had onchocerciasis disappear. Prevalence is among those aged 5+ years.

More on MDA strategies in case of two or three connected villages

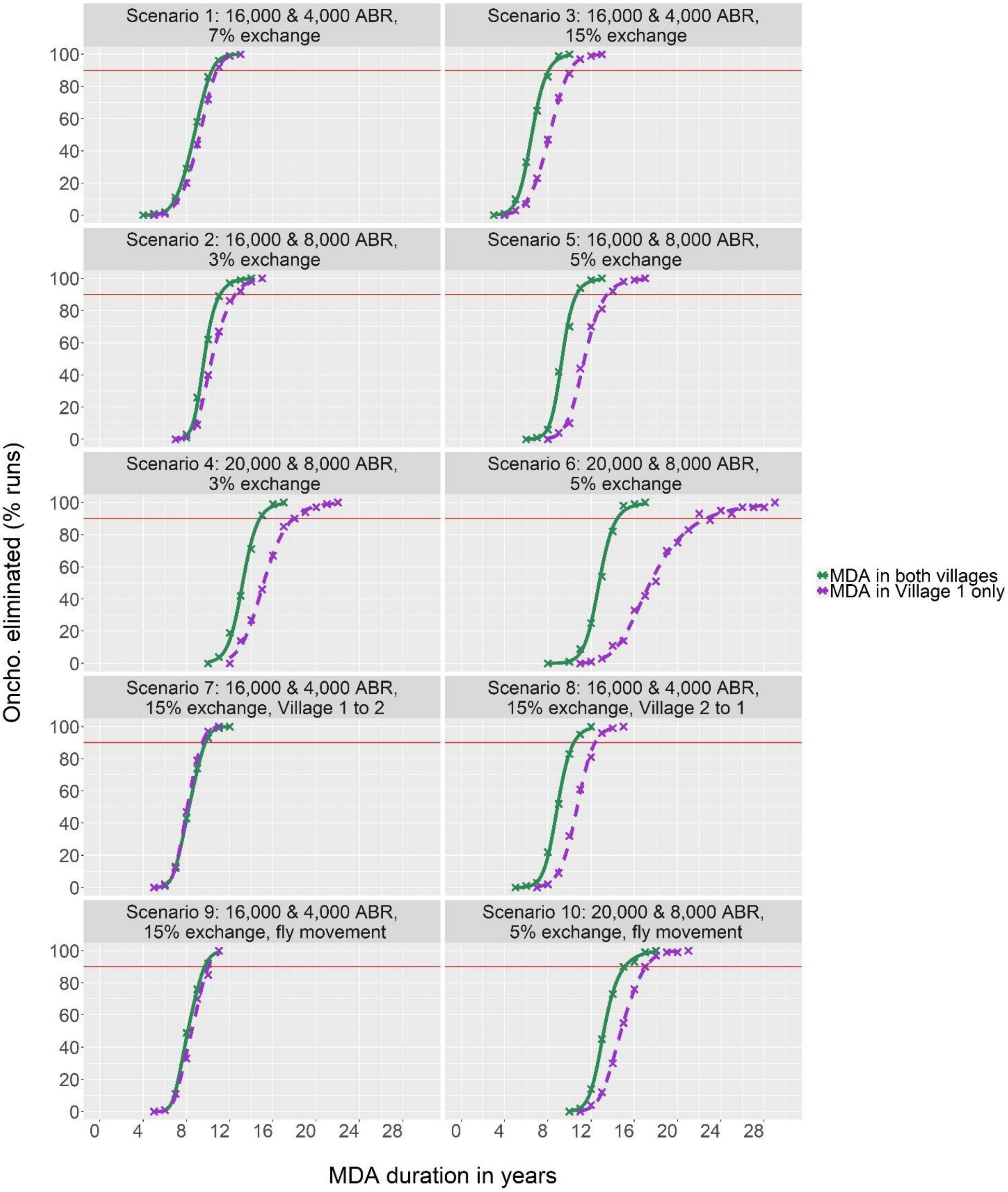

Fig F. Impact of MDA strategy on the probability of onchocerciasis elimination for different geographical settings. The two villages are connected as indicated in the panel headings, where not otherwise indicated, movement is symmetric and human (for more detailed scenario descriptions, see Table 1 (note, scenarios are here ordered by the ABRs rather than as in the main text Table 1, by the starting prevalence in the spill-over village(s)). Crosses represent the proportion of runs with onchocerciasis eliminated at 100 years post MDA; lines are fits to these points, see Methods.

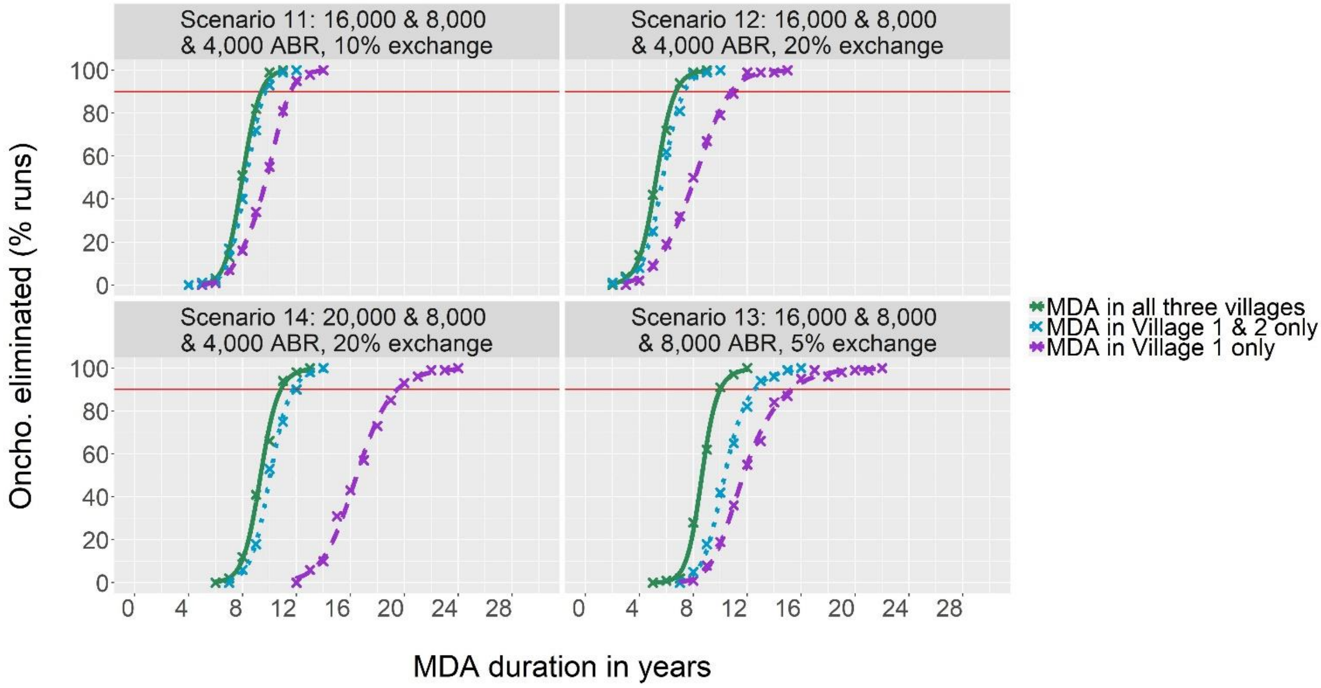

Fig G. Impact of MDA strategy in scenarios with three villages. In scenarios 11, 12 and 14, Village 3 is furthest from source Village 1, while in scenario 13, Village 2 and 3 are equally connected to Village 1. Crosses represent the proportion of runs with onchocerciasis eliminated at 100 years post MDA; lines are fits to these points, see Methods.

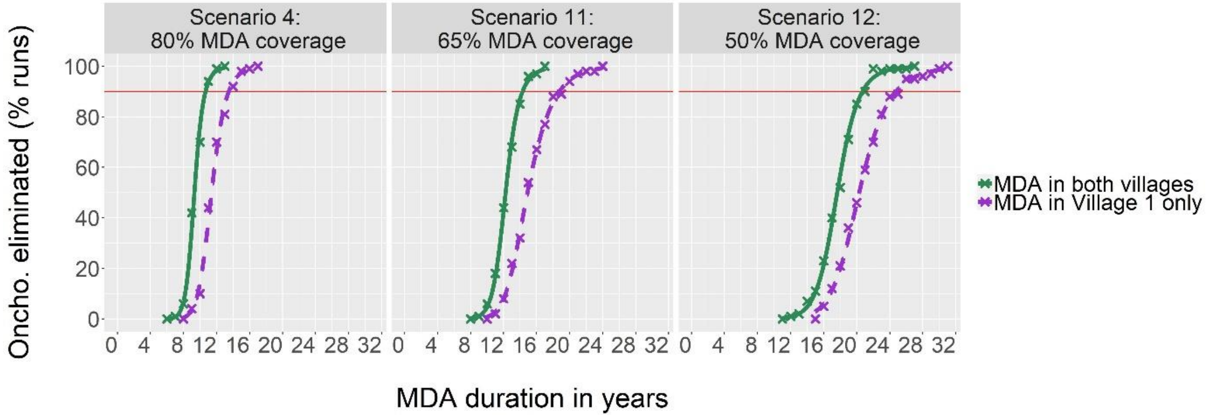

Fig H. Impact of MDA strategy in scenarios with different MDA coverages. For all three scenarios shown here, ABR Village 1 = 16,000, ABR Village 2 = 8,000, and individuals spent on average 5% of their time in the other village. Crosses represent the proportion of runs with onchocerciasis eliminated at 100 years post MDA; lines are fits to these points, see Methods.

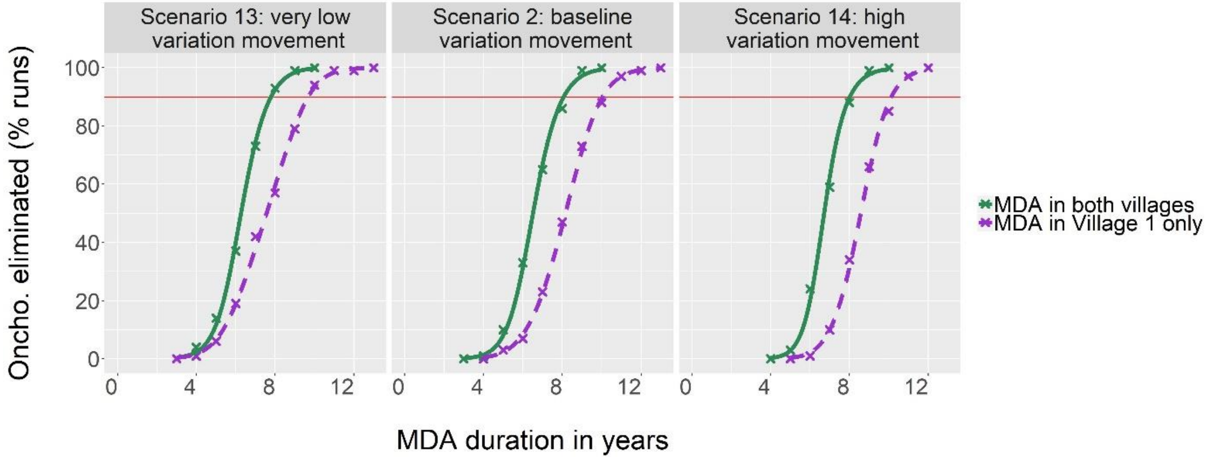

Fig I. Impact of MDA strategy in scenarios with different individual variation in the time spent outside the home village. For a description of this variation, see Fig E. For all three scenarios shown here, ABR Village 1 = 16,000, ABR Village 2 = 4,000, and individuals spent on average 15% of their time in the other village. Crosses represent the proportion of runs with onchocerciasis eliminated at 100 years post MDA; lines are fits to these points, see Methods.

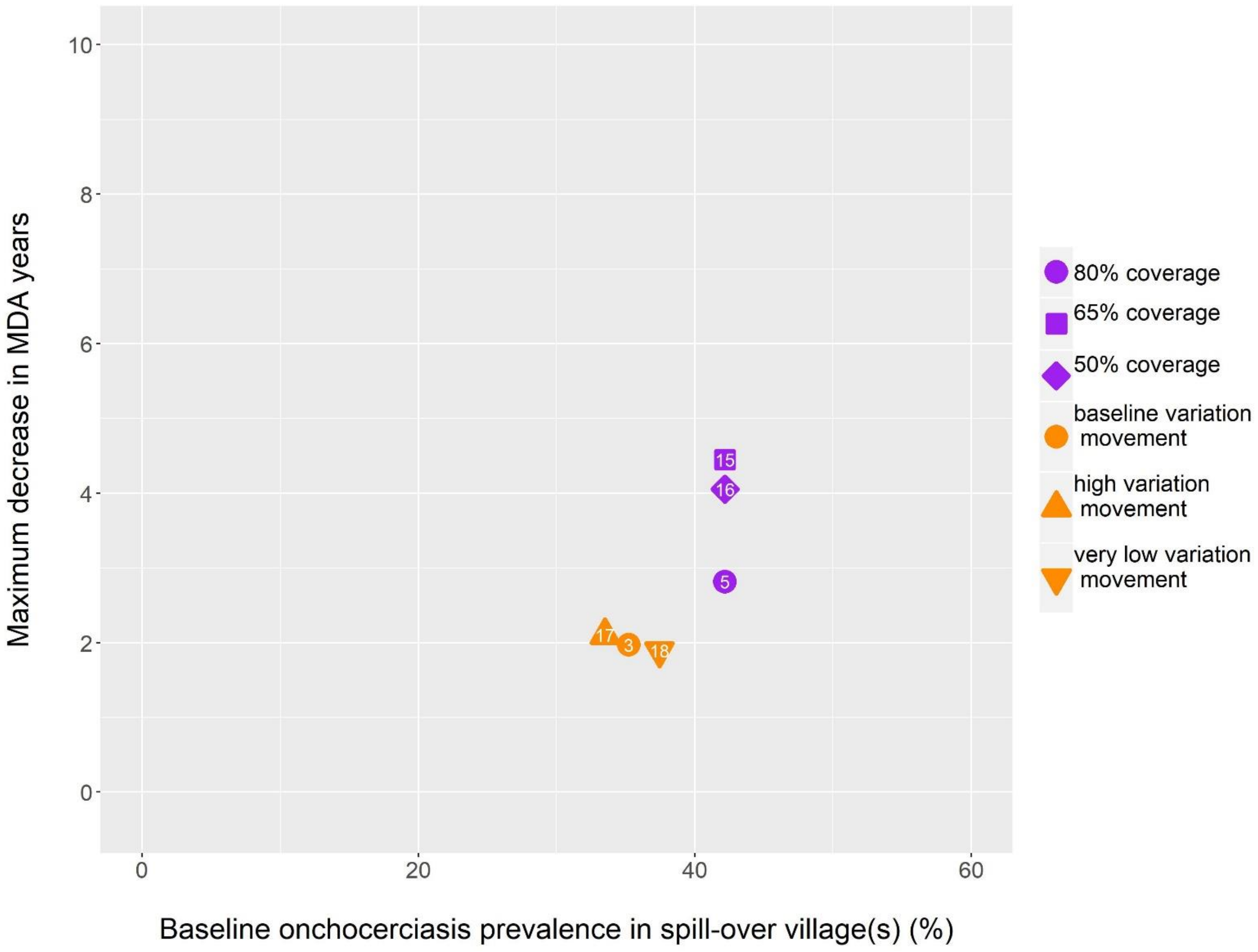

Fig J. Decrease in the number of years of MDA required for 90% onchocerciasis elimination probability, by treatment implemented throughout also in the spill-over prevalence village(s) rather than just in the higher prevalence village (parameter sensitivity scenarios). Numbers indicate the scenario settings, see Table 1 for details. Here we show the model outcome sensitivity to the MDA treatment coverage (% of inhabitants reached per MDA round), and the individual variation in the time villagers spend outside their home Village (see also Fig D). Prevalence is among those aged 5+ years.

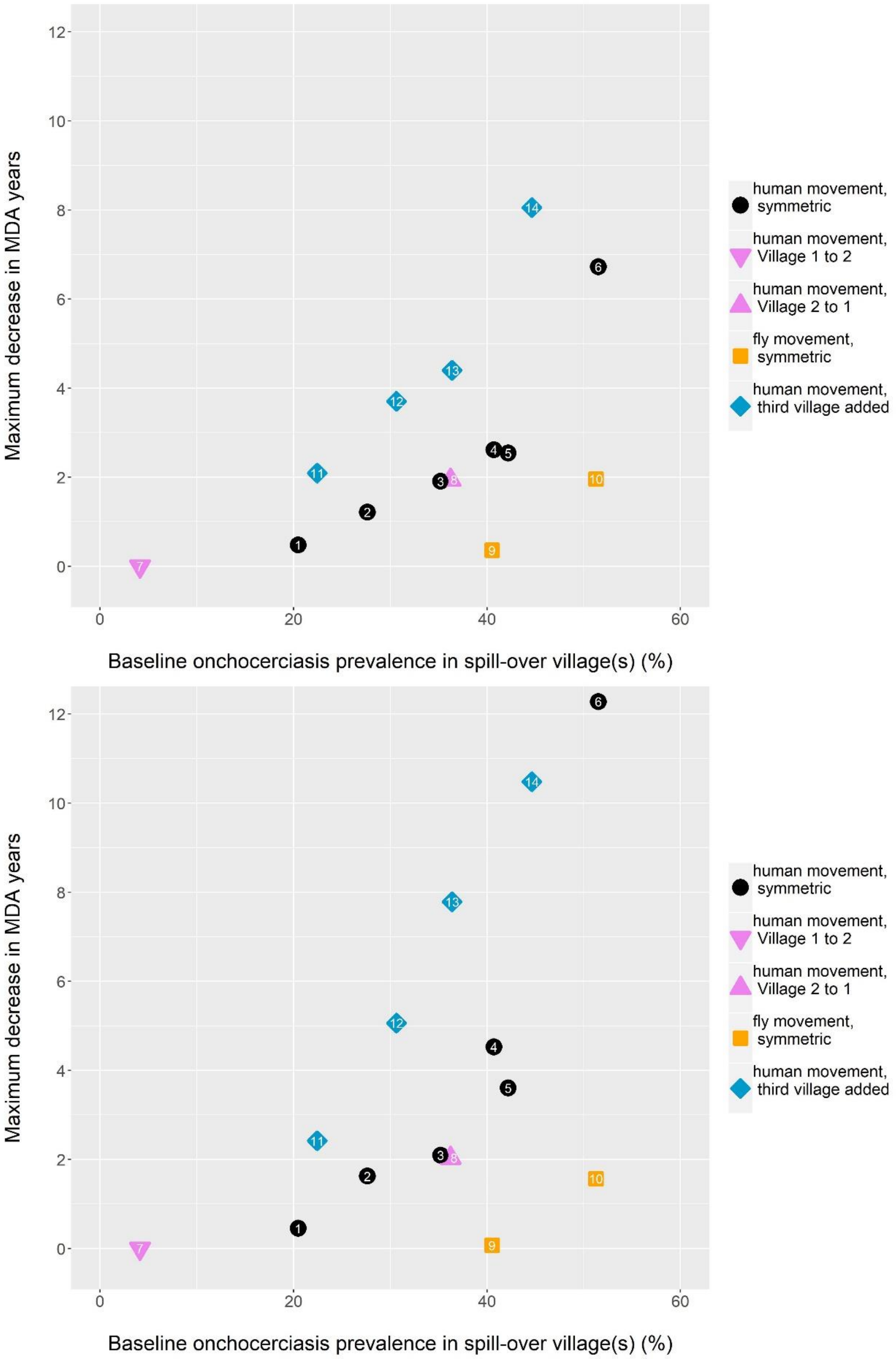

Fig K. Decrease in the number of years of MDA required for 80% (top) or 99% (bottom) onchocerciasis elimination probability, by treatment implemented throughout also in the spill-over prevalence village(s) rather than just in the higher prevalence village. Note that due to the limited number of 100 runs performed per setting per MDA strategy, measurement robustness is low for very high elimination certainties. Numbers indicate the scenario settings, see Table 1 for details.

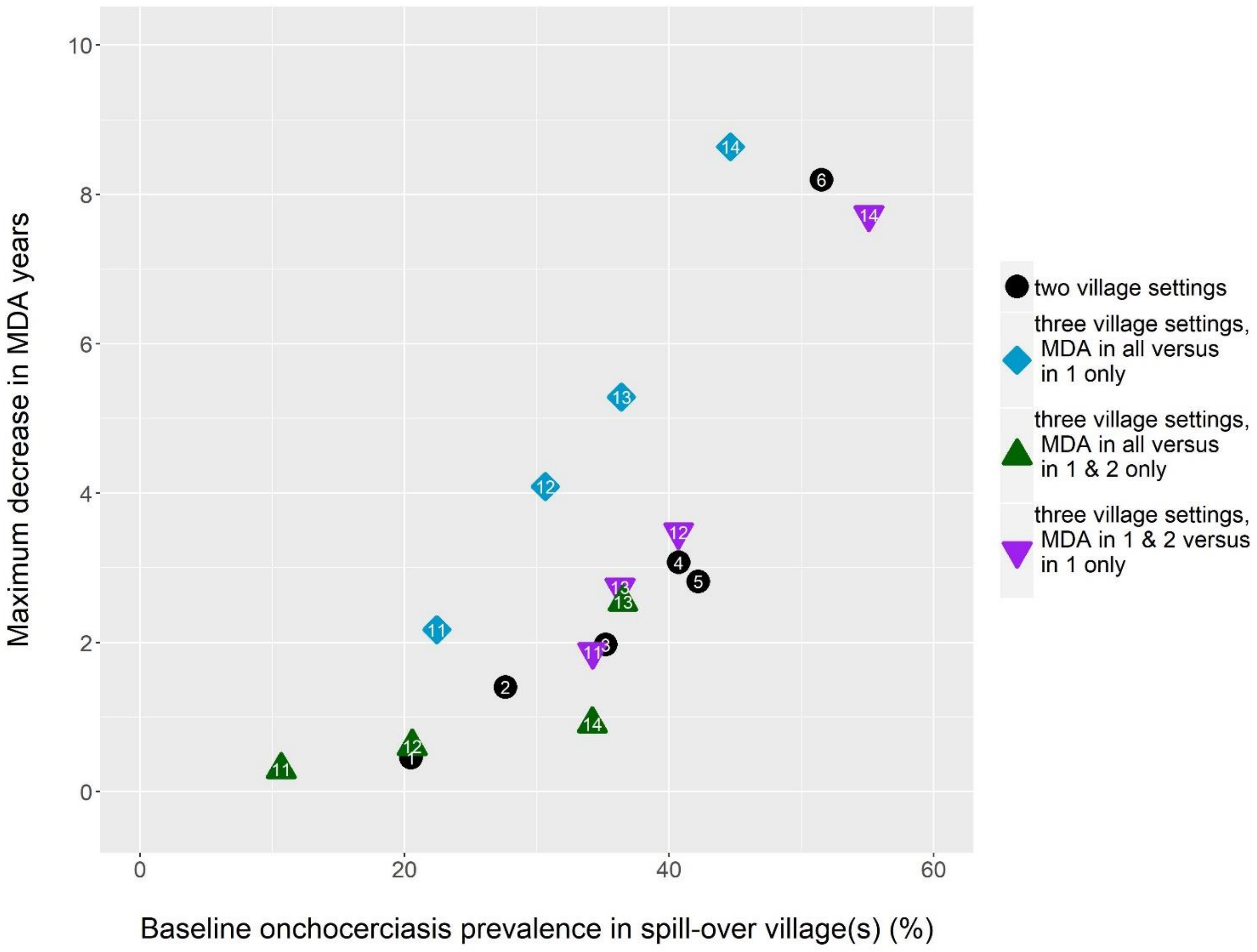

Fig L. Decrease in the number of years of MDA required for 90% onchocerciasis elimination probability, by treatment implemented throughout also in the spill-over prevalence village(s) rather than just in the higher prevalence village (with three village scenarios per village comparisons). Numbers indicate the scenario settings, see Table 1 for details.

For each three-village settings, we now make three comparisons, one for treatment implemented in Village 1 & 2 compared to implementation in Village 1 only, with prevalence in Village 2 on the x-axis (shown in purple triangles pointing down), second a comparison of implementing treatment in all three villages compared to in Village 1 & 2 only, with prevalence in Village 3 now on the x-axis (shown in green triangles pointing up), and third we compare implementing MDA in the total area to implementing it only in Village 1, with the mean prevalence of Villages 1 and 2 now on the x-axis (shown in blue diamonds). Prevalence is among those aged 5+ years.

On a per village basis, whether or not we add treatment of Village 2 or Village 3 respectively reduces the required number of years similarly as in the two-village setting, with the number of years depending on the prevalence in the village in question. However, if we consider Village 1 and Village 2 together as one spill-over location, and we use the overall prevalence in these two villages as a predictor for the use of treating them, we find approximately doubled impact. Clearly, a relatively more populous untreated prevalence spill-over location increases the risk for resurgence in the source village more. However, with too great connectedness to too large a location, stability of onchocerciasis prevalence in source Village 1 would be undermined (see Fig C).

More on MDA effects in case of unequal durations of MDA

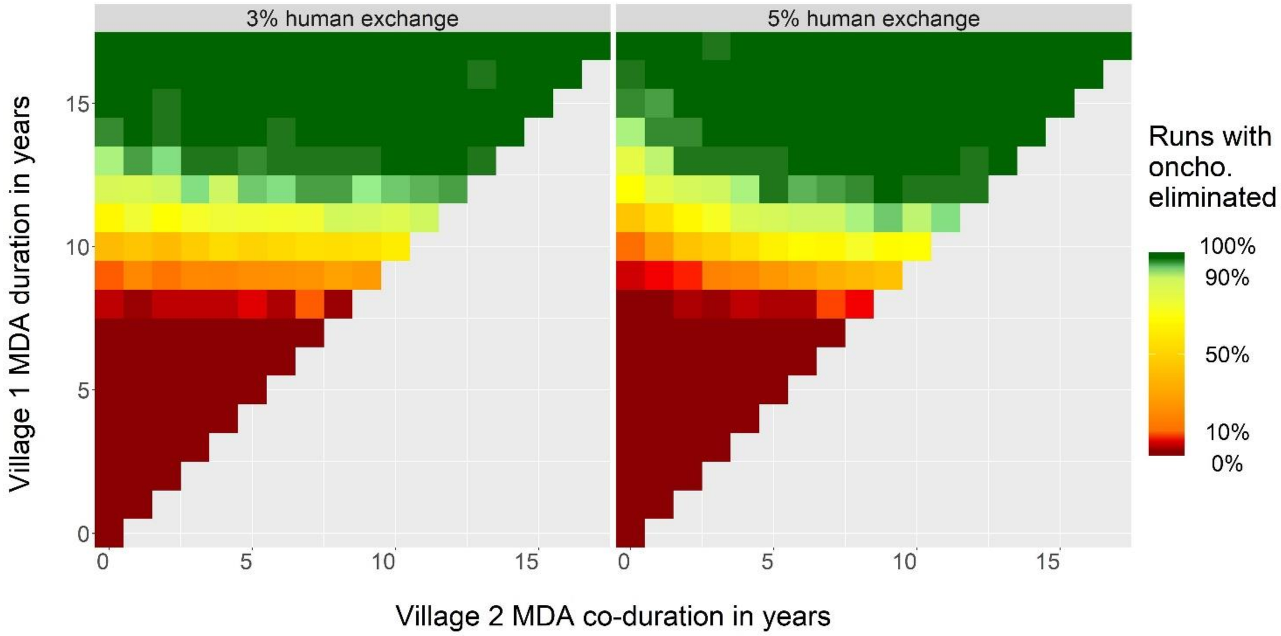

Fig M. Impact of MDA duration on onchocerciasis elimination when treating inhabitants of Village 2 fewer consecutive years than those in Village 1. MDA is started later in Village 2, so that the end year of MDA in the two villages is concurrent. 100 runs were performed per setting. ABR Village 1 = 16,000, ABR Village 2 = 8,000. On average, individuals spent 3 or 5% of their time in the other village respectively.

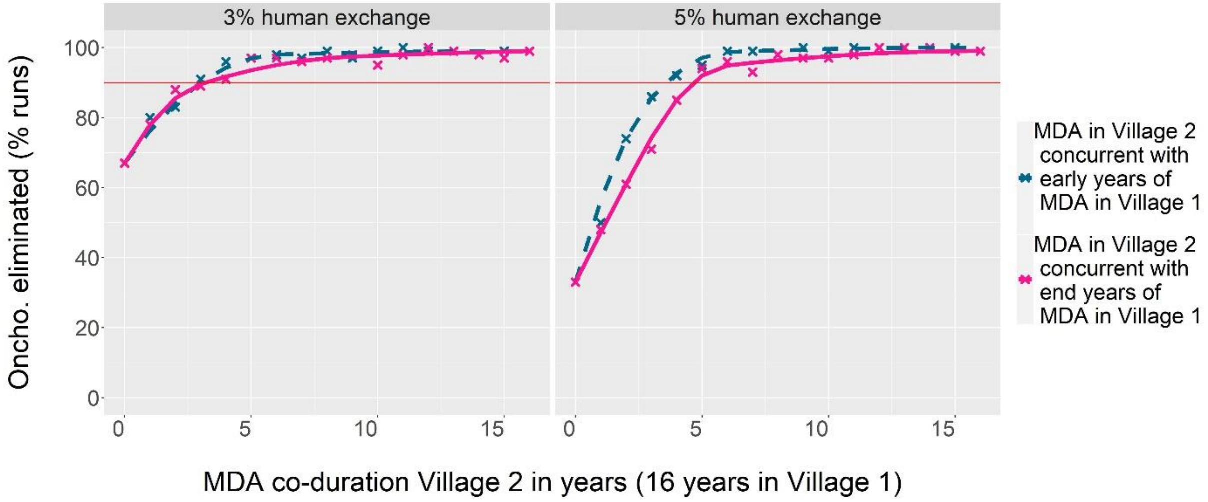

Fig N. Impact of MDA duration on onchocerciasis elimination when treating inhabitants of Village 2 fewer consecutive years than those in Village 1, as dependent on the timing of MDA in Village 2. ABR Village 1 = 20,000, ABR Village 2 = 8,000. On average, individuals spent 3% or 5% of their time in the other village respectively, i.e. these are scenarios 4 and 6 respectively, see main text Table 1. Crosses represent the proportion of runs with onchocerciasis eliminated at 100 years post MDA; lines are spline fits to these points.

In the geographical scenario settings considered here, pre-MDA prevalence is high; 87% in Village 1 for both scenarios, 41% or 52% respectively for 3% and 5% human exchange in spill-over region Village 2. In the first of these scenarios, if only implementing MDA in Village 1, it would take 18 years to reach 90% probability of onchocerciasis elimination (see main text Table 1), but the same could be reached by 16 years of MDA in Village 1 with 3 years of MDA in Village 2 (as shown here). In the 5% human exchange scenario, treating inhabitants of Village 2 can shorten the required MDA duration for obtaining 90% probability of onchocerciasis elimination more substantially, even rendering added treatment in Village 2 drug dose-saving; 90% probability of elimination could be reached by 24 years of MDA restricted to Village 1 (see main text Table 1), but 16 years of MDA in Village 1 with 5 years in Village 2 would also suffice (as shown here). Since the two villages have approximately the same number of inhabitants, the latter option would require less drugs.

For the same number of years that MDA is implemented also in Village 2, having the end years of treatment concurrent in the two villages will lead to a slightly higher probability for onchocerciasis elimination than having the start years concurrent. This as prevalence in Village 2 should be at its lowest when treatment in Village 1 ends, to obtain the smallest probability that contact with inhabitants of Village 2 will cause resurgence in Village 1.
