# Supplementary material for: The impact of mass drug administration expansion to low onchocerciasis prevalence settings in case of connected villages
Source: PLoS Negl Trop Dis. 2021 May 12;15(5):e0009011. doi: 10.1371/journal.pntd.0009011 (PMC8143415; doi:10.1371/journal.pntd.0009011)
Supplement: Attachment — Submitted filename: ResponseToReviewComments.docx [file pntd.0009011.s003.docx]

>> Dear editor,

We are grateful to the reviewers for their insightful remarks and their suggestions on improving our manuscript. Please find below our detailed response to their comments.

**Methods**

Reviewer #1: The study design is appropriate, even as additional clarity is needed in some areas.

Reviewer #2: The methods are clearly explained and described. The reasoning is sound and clear. It might help, perhaps in future work, to model differential MDA coverage or lower coverage in mobile groups. It may also help to note that identifying when and how far flies are traveling is difficult and resource intensive, but much easier to do so in humans.

>> Based also on the further advice of Reviewer #2 under Conclusions, and on advice of Reviewer #1 under Summary and General Comments, we have added a new paragraph to the Discussion on how we modelled fly-mobility, lines 446-454 of the tracked changes file:

“In this exploratory study, fly movement was modelled in a relatively simple manner, migrating a fraction of infected flies directly from one location to another each month. We did not consider the possibility of excess mortality among migrating flies, nor possible associations between migration, fly age and infection status [17]. By ignoring these finer points, we may have somewhat overestimated the potential for fly movement to cause spill-over prevalence. Measuring how far flies tend to travel is difficult and labour-intensive [17], making it hard to identify realistic ranges for the fraction of flies migrating. Since we found that extending MDA to spill-over locations had only limited impact on the required MDA duration for the total area when the spill-over was due to fly movement, we chose to explore mostly MDA scenarios with villages connected by human mobility.”.

In the following paragraph of the Discussion (lines 455-468), we now discuss the option for future work as suggested here by Reviewer #2:

“Human mobility was simulated assuming that individuals spend a limited portion of their time outside of their home village each month. Although human movement would be easier to study than fly movement, it is also generally undocumented, making it hard to quantify more elaborate mobility models. Our model could be extended to examine other forms of connection by human mobility, such as by seasonal or long-term human migration. We found that the total amount of connection by mobility between locations, rather than the individual movement patterns of inhabitants, determines spill-over and probability for resurgence. Also given the 10-year average lifespan of worms, we therefore do not expect that seasonal worker migration instead of the constant commuting rate considered here would lead to substantially different results. However, in our analyses we assumed that the individual human movement rate was not associated with MDA participation, while those spending much time away from their home village could be less likely to participate in local MDA. This would represent a form of systematic individual non-participation, as has been considered in other ONCHOSIM studies [7]. Specifically targeting migrant workers could then be considered as a treatment strategy to prevent resurgence.”

**Results**

Reviewer #1: The analysis is robust and the results clearly presented.

Reviewer #2: The results are generally clearly presented and framed in such a way that they will be useful for programs. Question: in lines 272-275, the text describes an average of 28% mf prevalence in lines showing elimination. This is confusing - shouldn't prevalence be much, much lower if elimination is achieved? Question: in lines 361 to 363, the text says that "..the required MDA duration when treating only Village 1 is always less than double (or triple) that of throughout treating both (or all three) villages..." Is this not backwards? Isn't the duration of treatment longer when focused only on Village 1?

>> For Village 2, with relatively low fly density, where transmission would not be self-sustaining without influx of infection through human or vector mobility, we do see a high likelihood of *eventual* elimination, even with 28% prevalence remaining here when MDA is stopped in source Village 1. In the sentence referred to by the Reviewer starting at line 276, we have added a few words to make this clearer:

“With MDA implemented in just the source location (Village 1), resurgence is to a large extent determined by the onchocerciasis prevalence in the spill-over prevalence location (Village 2) at the end of MDA; for those 30 runs showing resurgence, the mean prevalence in Village 2 at the end of MDA (vertical lines at 12 years) is 35% (range 21%-48%), while it is on average 28% (14%-44%), among the 70 runs showing eventual elimination.”

We have fully replaced the sentence the reviewer here points to as confusing, at line 370 we now state:

“Since the modelled villages have equal population sizes, expanding treatment throughout to Village 2 (and 3) would only be drug-dose saving if the total number of required MDA rounds would then be less than half (or a third) of that needed for treating only Village 1. This is not the case in any of the considered settings (Table 1).”

**Conclusions**

Reviewer #1: The authors' conclusions are supported by the data presented.

Reviewer #2: The conclusions relate directly to the data and anticipate many questions from readers. The conclusions seem to focus more on human populations, whose movement is easier to monitor and evaluate.

>> See also our reply above, under Methods. We have added to the Discussion an explanation for our focus on human movement:

“Since we found that extending MDA to spill-over locations had only limited impact on the required MDA duration for the total area when the spill-over was due to fly movement, we chose to explore mostly MDA scenarios with villages connected by human mobility.”

**Editorial and Data Presentation Modifications**

Reviewer #1: (No Response)

Reviewer #2: For all this paper's strengths, it relies on parasitological measures and levels of prevalence that are, nowadays, not as commonly encountered in active programs. Current guidelines for interruption of transmission rely on serological and entomological endpoints, which likely correlate with mf prevalence far below that studied in this paper and those recommended by APOC. It is also known that lower levels than 20-40% mf exist and persist in absence of a traditionally hyper-endemic village nearby. Finally, programs with ongoing MDA may have suppressed their mf levels below the thresholds in this paper, but could still benefit from these results. In this reviewer's opinion, it would be helpful to address 1) how this work could be validated or supported with other indicators of transmission, and 2) present ideas for programs to utilize this approach when they either do not have baselines or have undertaken several years of MDA.

>> We have added to the Discussion (lines 505-516) a paragraph addressing both questions:

“Demonstrating dependency on neighbouring high-transmission settings in the field would be useful to guide local elimination policy in specific situations, and to validate our work, but is challenging. Information can be obtained from long-term follow-up studies tracking changes in prevalence in neighbouring treated and untreated communities: in case of dependency, a decline in prevalence would be expected in the untreated village, as shown in Figure 4, bottom-left. Since skin snips studies on mf levels are now less common, for a comparison with such field data, future modelling work should include other infection indicators such as OV-16 antibody prevalence in children [23], or infection in flies. Additional information on general levels of connectedness can perhaps also come from large-scale genetic studies of worms and flies [9] or by studying the role of infection import in settings with post-MDA resurgence. Quantifying the degree of connectedness in specific settings will usually be difficult due to uncertainty about local transmission conditions, baseline endemicity and exact history of control.”

**Summary and General Comments**

Reviewer #1: This is a well-written, rigorously designed study that uses a mathematical model to explore the impact of the degree/direction of population connectedness on onchocerciasis transmission, as well as the effect of local vs. large-scale mass drug administration across a set of connected communities. The authors find that—in setting below the ABR threshold needed for stable local transmission—persistence of onchocerciasis can be explained by population movement (both human and intermediate hosts) from areas with higher ABR. They examine the theoretical constraints on elimination of onchocerciasis when MDA in undertaken in the higher prevalence, contributing area. A major strength is the formal expression and estimation of shortened MDA requirements when target populations are expanded to include the lower ABR areas. Some questions are raised over the treatment of movement in the model—the treatment of fly host movement, for instance, is not clear. What is the modeled (or implied, based on the representation in the model) time course/rate of movement? Given the short lifespan of the host, how might the effects of movement be counteracted by mortality during/following movement?

>> See again also our first reply under Methods, in our new Discussion paragraph we state: “In this exploratory study, fly movement was modelled in a relatively simple manner, migrating a fraction of infected flies directly from one location to another each month. We did not consider the possibility of excess mortality among migrating flies, nor possible associations between migration, fly age and infection status [17]. By ignoring these finer points, we may have somewhat overestimated the potential for fly movement to cause spill-over prevalence.”

Over what time period does the convergence of V1 and V2 prevalences occur (across increasing degrees of movement) in Figs 2, 3, and might the time-to-convergence be relevant to consider with respect to adaptive MDA strategies, as well as the possibility that seasonal movements may ameliorate or exacerbate the convergence?

>> As stated in our Methods section line 146-147 “. In our current investigation, we allow only stable transit (i.e., constant throughout a simulation) of humans or flies between villages.” That is, we assume the movement is long-standing, so that prevalences of the different villages have converged before MDA starts. Technically, we used a burn-in period of 120 years for each model run to obtain this equilibrium situation. See also our first reply under Methods, where we quote our addition to the Discussion on seasonal migration.

Which regions on the x-axis of figure 3 (bottom panel) are realistic/meaningful given actual host movement behavior? I understand the researchers are seeking a highly simplified model system in order to explore the effects of movement, but doing so with more robust treatment of movement dynamics may yield greater insights.

>> As stated in our new paragraph on fly movement in the Discussion, see our first reply under heading Methods: “Measuring how far flies tend to travel is difficult and labour-intensive [17], making it hard to identify realistic ranges for the fraction of flies migrating.”

Another area of concern is the potential for heterogeneity in human host movement behaviors to upend the findings of the study. It is not clear why, if movement is confined to a small number of highly mobile individuals (as is observed in many contexts), the qualitative results of the study do not change (as represented in Fig S5)? Deeper explanation and exploration is needed here, in this reviewer’s view, to make a more convincing case that heterogeneity is unimportant given prior research on connectivity and movement heterogeneity.

>> We have added exploration of an even more extreme case of human movement heterogeneity, see the new Supplement Figure S5, where we also show for more situations how the model implements the variation over the modelled village population.

We have added explanation to the text of the Results section at line 250, where we refer to the Supplement Figure S5:

“We also considered different degrees of heterogeneity between individuals in their tendency to go outside of their home village (Figure S5), and find this factor not to have much effect: it is the total amount of connection between the village clouds of intermediate hosts, rather than individual movement behaviour that causes spill-over of prevalence.”

At line 335, we have expanded our statement on the effect of human movement heterogeneity on our MDA results:

“The risk for recrudescence in Village 1 to be triggered from Village 2 is only very modestly impacted by variation among villagers in their tendency to go outside of their home village (Figure S10), again indicating that it is the overall degree of connectedness by mobility rather than individual movement patterns that matter.”

Reviewer #2: This paper will be important for the onchocerciasis community, as it quantifies the effects of human and fly movement on transmission. Although it would make programs easier, humans and vectors do not simply stay in one place for the duration of an elimination program. They move, and this has effects both on their home communities as well as others. MDA programs should be sensitive to these realities but often lack the tools and frameworks to understand the implications.

The traditional monitoring approach is to focus on specific (usually highest-prevalence) villages; nearby villages are either investigated as well - which can be resource intensive - or the results from the highest risk village are assumed to be true for other areas. In the worst case scenario, nearby or farther flung villages are ignored entirely, although it is clear that humans, and possibly flies, are quite comfortable moving between them. This limits the effectiveness of MDA programs. MDA can go on longer than needed, or communities are at risk of recrudescence due to previously ignored populations.

>> We certainly agree with the reviewer.
